# Disturbance history mediates climate change effects on subtropical forest biomass and dynamics

**DOI:** 10.1002/ece3.5289

**Published:** 2019-05-27

**Authors:** Alexandre F. Souza, Solon Jonas Longhi

**Affiliations:** ^1^ Programa de Pós‐Graduação em Ecologia, CB Universidade Federal do Rio Grande do Norte Natal Brazil; ^2^ PPG Engenharia Florestal, Depto. Ciências Florestais Universidade Federal de Santa Maria Santa Maria Brazil

**Keywords:** *Araucaria angustifolia*, Atlantic Forest, Brazil, climate change, community composition, forest biomass, forest dynamics, functional traits, logging, subtropical forests

## Abstract

The responses of forest communities to interacting anthropogenic disturbances like climate change and logging are poorly known. Subtropical forests have been heavily modified by humans and their response to climate change is poorly understood. We investigated the 9‐year change observed in a mixed conifer‐hardwood Atlantic forest mosaic that included both mature and selectively logged forest patches in subtropical South America. We used demographic monitoring data within 10 1 ha plots that were subjected to distinct management histories (plots logged until 1955, until 1987, and unlogged) to test the hypothesis that climate change affected forest structure and dynamics differentially depending on past disturbances. We determined the functional group of all species based on life‐history affinities as well as many functional traits like leaf size, specific leaf area, wood density, total height, stem slenderness, and seed size data for the 66 most abundant species. Analysis of climate data revealed that minimum temperatures and rainfall have been increasing in the last few decades of the 20th century. Floristic composition differed mainly with logging history categories, with only minor change over the nine annual census intervals. Aboveground biomass increased in all plots, but increases were higher in mature unlogged forests, which showed signs of forest growth associated with increased CO_2_, temperature, and rainfall/treefall gap disturbance at the same time. Logged forests showed arrested succession as indicated by reduced abundances of Pioneers and biomass‐accumulators like Large Seeded Pioneers and Araucaria, as well as reduced functional diversity. Management actions aimed at creating regeneration opportunities for long‐lived pioneers are needed to restore community functional diversity, and ecosystem services such as increased aboveground biomass accumulation. We conclude that the effects of climate drivers on the dynamics of Brazilian mixed Atlantic forests vary with land‐use legacies, and can differ importantly from the ones prevalent in better known tropical forests.

## 1 INTRODUCTION

Disturbance is a natural process that occurs at different spatial and temporal scales (Pickett, Kolasa, Armesto, & Collins, 1989). It plays a key role in the evolution and maintenance of plant ecological strategies (Grime & Pierce, ) and is often considered a critical element in maintaining native plant communities (Davies, Svejcar, & Bates, 2009). At a local scale, forest disturbance may seem relatively straightforward and well‐studied, since the death of one or several trees creates canopy gaps that trigger small‐scale forest succession, whose trajectory is largely dictated by functional trait distribution across species (Lohbeck et al., 2014; Muscolo, Bagnato, Sidari, & Mercurio, 2014). However, it has been increasingly recognized that prior disturbances exert a strong influence on the response of plant communities to subsequent disturbances (Davies et al., 2009). Forest response to the interaction of different anthropogenic disturbances like logging and climate change is still poorly understood. In this study, we examine the importance of different types of disturbance for forest composition, structure, and biomass levels in subtropical mixed conifer‐hardwood Atlantic forests in South America.

Results from a variety of studies point toward global increased tree growth and accelerating tropical forest dynamism, leading to forests with increasing biomass and carbon storage (Castanho et al., 2015; Chave et al., 2008; Holtum & Winter, 2010; Lewis, Lloyd, Sitch, Mitchard, & Laurance, 2009; Pan et al., 2011; Phillips, Lewis, Higuchi, & Baker, 2016) but see (van der Sleen et al., 2014). The ubiquity of these changes (Pan et al., 2011) suggests that its main drivers be long‐term climatic change like atmospheric CO_2_ concentrations and precipitation patterns (Phillips et al., 2016), although other drivers may also play a role (reviews in (Lewis, Malhi, & Phillips, 2004; Körner, 2009; Lewis et al., 2009; Phillips et al., 2016). Forest responses to climate change, however, may interact with recovery from past disturbances such as logging (Clark, 2007; Enquist & Enquist, 2011). Unsustainable selective logging is capable of producing long‐lasting functional changes in forest trait distribution (Both et al., 2019), the collapse of logged populations (Ter Steege, Welch, & Zagt, 2002), and arrested succession (Chazdon, 2003; Edwards, Tobias, Sheil, Meijaard, & Laurance, 2014; Souza, Cortez, & Longhi, 2012) but see (Pyles, Prado‐Junior, Magnago, de Paula, & Meira‐Neto, 2018). Together, both climate change and logging may change forest dynamics and functioning through changes in forest structure, species richness, floristic composition, species ecological strategies, and functional trait diversity (Díaz & Cabido, 2001; Enquist & Enquist, 2011; Grime & Pierce, ; Poorter et al., 2017; Pyles et al., 2018; van der Sande et al., 2016).

Related knowledge gaps include strong biases toward old‐growth forests (Longo & Keller, 2019) and the drivers of the marked regional differences in forest change that have been registered with some regions, often wet ones, presenting biomass gains while others, often drought‐prone ones, presenting losses (Castanho et al., 2015; Shen et al., 2013; Stephenson & Mantgem, 2011; van der Sande et al., 2016). The long‐term effects of human activities like logging also need further understanding, since most studies have taken place shortly after the impact and may conceal low decays of biodiversity or ecosystem function (Edwards et al., 2014). Specifically, we tested the hypothesis that climate change affected forest structure and dynamics differentially depending on past disturbances represented by logging history. We expected that climate change at both global and regional scales affects forest dynamics. Both experiments and theory indicate that plant photosynthesis should increase in response to globally rising CO_2_ concentrations, leading to increased plant growth and forest biomass (Lewis et al., 2009, 2004). Rising CO_2_ concentrations are accompanied by rising temperatures (IPCC, 2013). In the subtropics, growth is limited by winter low temperatures (Oliveira, Roig, & Pillar, 2010; Zanon & Finger, 2010). Thus, subtropical mixed forest biomass accumulation should be particularly promoted by global CO_2 _buildup and warming. A second mechanism leading to biomass accumulation in mixed forests is niche complementarity between angiosperms and conifers. Complementarity refers to niche differences between species or species groups that, due to the possession of distinct sets of traits, explore available resources in different ways and thus increase ecosystem performance, like biomass accumulation (Loreau & Hector, 2001).

We expected forest biomass, basal area, tree density, and tree turnover to increase in all forest patches due to the long‐term and pervasive effects of increased CO_2_ concentration and temperature. This increase, however, should be more pronounced in logged stands, in which climatic effects were combined with recovery from past disturbance. These increases should be higher also among Small Shade Tolerant species, which form the most light‐ and CO_2_‐limited ecological group (Falster, Duursma, & FitzJohn, 2018; Körner, 2009; Malizia, Easdale, & Grau, 2013; Zuidema et al., 2013). Due to CO_2_‐based increase in shade tolerance, we expected decreases in unlogged plots in the community‐weighted means (CWM) of leaf size, specific leaf area, tree height, stem slenderness, and wood density, while crown depth and seed size should increase.

Increased rainfall in southern Atlantic forests is regionally accompanied by more frequent tornados and windstorms, which peak in the subtropics due to rainier climate and colliding cold and hot air masses (Candido, 2012). This implies both reduced solar radiation due to increased cloudiness and increased small scale secondary succession due to treefall gap dynamics (Ge, Xiong, Zhao, Shen, & Xie, 2013). Reduced solar radiation limits primary production (Graham, Mulkey, Kitajima, Phillips, & Wright, 2003, but see Mercado et al., 2009), while disturbance by treefall gap dynamics triggers small scale succession (Ge et al., 2013; Muscolo et al., 2014). We expected that in unlogged plots mortality would be the highest among the tallest trees (Beckert, Rosot, & Rosot, 2014; Ebling, Guimarães, Pelissari, Abrão, & de Miranda, 2014), triggering increases in pioneer species due to increased regeneration opportunities in treefall gaps (Chami, Araujo, Longhi, Kielse, & Lúcio, 2011). If logged forests are recovering from past human disturbances, we expected an increased representation of slow‐growth shade‐tolerant species and of long‐lived late‐successional species (Edwards et al., 2014; Hogan et al., 2018; Malizia et al., 2013). These functional groups are expected to replace fast‐growing and short‐lived species that characterize disturbed forests (Rozendaal & Chazdon, 2015). Additionally, we expected that the abundance of the long‐lived pioneer groups Large‐Seeded Pioneers and Araucaria should increase in recently logged stands, but not in unlogged and early logged stands. Long‐lived pioneers attain large adult sizes but depend on large canopy openings for successful regeneration, and the closed‐canopy structure of unlogged and early logged stands should thus prevent these group’ regeneration to the adult size class (Souza, 2007; Souza, Forgiarini, Longhi, & Brena, 2008). We also expected that in logged forests the CWM of leaf size, specific leaf area, and crown depth would decrease, while those of wood density, stem slenderness, seed size, and tree height should increase, reflecting a successional change toward more mature forests (Edwards et al., 2014; Hogan et al., 2018; Malizia et al., 2013). As a consequence, we expected tree density to decline (Rozendaal & Chazdon, 2015), while basal area and biomass would increase (Pyles et al., 2018), since these features are largely dependent on the presence of thick, tall trees with high wood density (Conti & Díaz, 2013; Edwards et al., 2014; Lindenmayer & Laurance, 2017; Poorter et al., 2017; Slik et al., 2013). If the above‐mentioned changes also occurred with less intensity in unlogged patches, this would suggest forest recovery from older disturbances, apart from known management history.

## 2 MATERIAL AND METHODS

### 2.1 Study area and data collection

**Figure 1:**
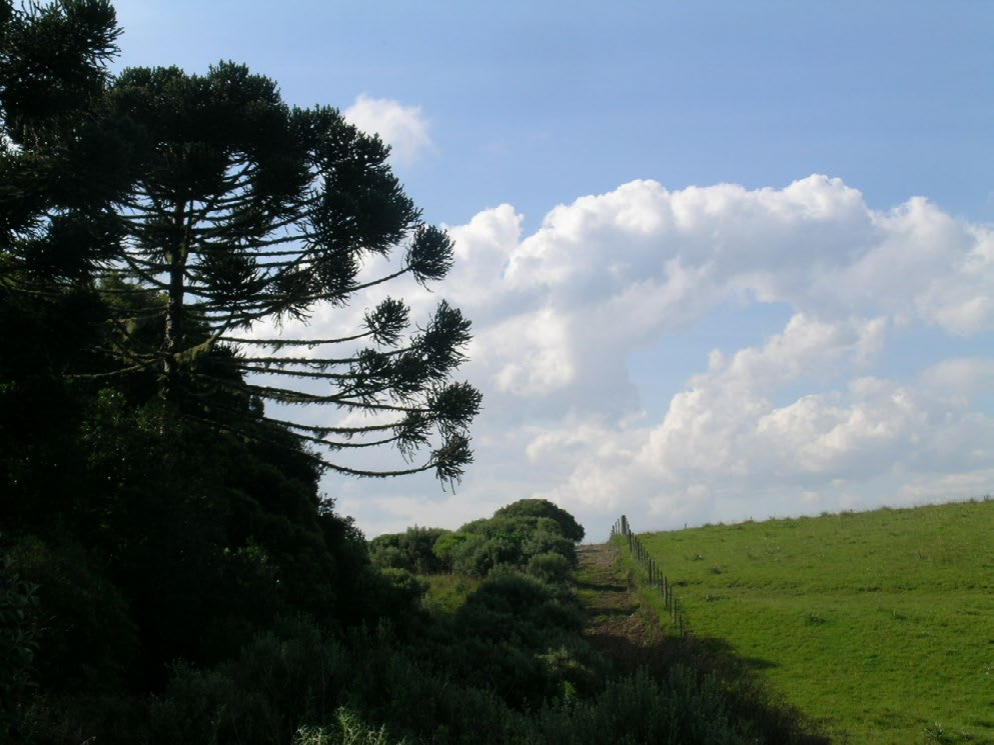
A recently logged forest plot near the edge of the São Francisco de Paula National Forest. Neighboring grasslands and an emergent *Araucaria angistifolia* are visible

The studied forests are located in the São Francisco de Paula National Forest, Rio Grande do Sul state, Southern Brazil (29°25′S, 50°23′W). The studied forests are diversity‐rich Atlantic forests (Souza et al., 2012) and represent a floristic transition between the tropical floras of eastern South America and the temperate floras of the southern parts of the continent (Eastern Mixed Forests sensu Gonçalves & Souza, 2014). The National Forest consists of 1,600 ha and contains most of the tree diversity present in the heavily logged and grazed region (Souza et al., 2012). The forests are dominated by *Araucaria angustifolia*, a long‐lived pioneer species that establishes in seminatural grasslands or in forest edges and multiple treefall gaps after large‐scale disturbances, but fails to regenerate underneath the closed canopy formed by angiosperms (Souza, 2007, 2017; Souza et al., 2008) (Figure 1). Climate is Köppen–Geiger type Cfb, which is humid subtropical with temperate summers with no true dry season (Alvares, Stape, Sentelhas, de Moraes Gonçalves, & Sparovek, 2013). Monthly temperatures remain under 15°C for up to 8 months of the year and mean annual rainfall is 2,252 mm. Soils are derived from volcanic rocks and altitude is ca. 900 m. Although Southern Brazil in general has not experienced significant mean temperature changes in the last century (Rosenblüth, Fuenzalida, & Aceituno, 1997), the annual frequency of cold nights has diminished (IPCC, 2013). The region experiences strong ENSO effects, with excess rain (including extreme floods) and temperatures during El Niño years, and droughts and cooler temperatures during La Niña years (Garreaud, Vuille, Compagnucci, & Marengo, 2009; Grimm, Ferraz, & Gomes, 1998). Furthermore, rainfall has consistently increased in southern South America during the second half of the XXth century due to more El Niño‐dominated conditions (Haylock et al., 2006), with the greatest increases occurring in Southern Brazil (Penalba & Robledo, 2010).

Data were collected in 10 100 × 100 m (1 ha) plots, covering local (1–10 km) and habitat (10–1,000 m) scales. Five of the studied plots had never been logged (hereafter unlogged plots), while the other plots experienced uncontrolled selective logging until 1955 or 1987 (hereafter early‐logged and recently logged plots, respectively). A detailed analysis of the plots and their histories can be found in Souza et al. (2012), and an overall presentation of species changes can be found in Ebling et al. (2014). Plots were originally laid down avoiding steep slopes and water courses, and were thus physiographically similar (Souza et al., 2012). In 1999–2000, all tree species with dbh ≥ 9.5 cm were tagged and identified to species level, totaling 7,979 trees, distributed in 148 species, 91 genera, and 46 botanical families. Trees were remeasured for survival, ingrowth (new trees with dbh ≥ 9.5 cm), and dbh annually from 2001 to 2009. In 2010, data on total height, stem slenderness, crown depth, wood density, specific leaf area, leaf and seed length were measured for the 66 most abundant species. Stem slenderness was the species‐specific expected tree height for a standard dbh of 15 cm and was regarded as a measure of light‐demanding ecological strategy (Poorter, Bongers, Sterck, & Wöll, 2003). Based on these multi‐trait data, (Souza, Forgiarini, Longhi, & Oliveira, 2014) statistically identified six functional groups of tree species: Araucaria, Pioneers (fast‐growing, small‐seeded, light demanding), Large Seeded Pioneers (fast‐growing, large‐seeded, light demanding), Wind‐Dispersed Large Trees (tall trees, wind‐dispersed trees, slow growing), Large Shade Tolerants (tall trees, slow‐growing, shade‐tolerant), and Small Shade Tolerants (slow‐growing, shade‐tolerant treelets).

### 2.2 Data analysis

#### 2.2.1 Climate trends

We evaluated temperature and rainfall records for the study area in search of long‐term local climate changes. We used a 1901–2014 data set from the Climate Research Unit TS 3.23 database (CRU, Harris, Jones, Osborn, & Lister, 2014), which contains gridded global climate data from monthly observations interpolated into 0.5° latitude/longitude grid cells. All statistical analyses were carried out in the R 3.4.3 statistical software (R Core Team, 2017). LOESS regressions (Jacoby, 2000) were fitted to all time series to check for the presence of nonlinear patterns. Autocorrelation of the residuals from fitted linear regressions of climatic variables on time was tested by with the Durbin–Watson test using the Durbin–Watson test function in the “car” 2.1.3 package and by autocorrelation function plots using the acf function in the “stats” base package. To assess the accuracy of this global data set for the studied forests, we compared it to annual rainfall data from the Passo Tainhas meteorological station in São Francisco de Paula (data available from 1945 to 2006). Since the correlation of annual rainfall between the meteorological station and the CRU data set was high (*r* = 0.718, *p* < 0.0001), the CRU data were used to assess temporal trends in annual rainfall, and highest and lowest minimum annual temperatures (referred to as summer and winter minimum temperatures, respectively). We decided to evaluate minimum temperature trends since low air temperature may be a limiting factor for photosynthesis (Lewis et al., 2013), and because the annual frequency of cold nights has diminished in Southern Brazil during the last century (IPCC, 2013). Outliers were searched for using the grubbs.test function in the “outliers” 0.14 package with option type = 11, which tests if the lowest and highest values are two outliers on opposite tails of the sample. Two outliers were detected for the rainfall series. However, we decided to keep them in the analyses because removing them did not alter the results in any meaningful way.

#### 2.2.2 Species composition and ecological groups

For analyses of forest stands, trees were grouped into 20 × 20 m subplots, and analyses were restricted to the overall 2000–2009 interval. We used a nested PERMANOVA (Anderson, 2001) to test for the effects of time (a factor with two levels) and management history (a factor with three levels) on species abundances at the plot level. The analysis was performed using the function adonis in the “vegan” 2.4‐6 package (Dixon, 2003), with the Bray–Curtis distance and 5,000 permutations. One‐ha plots were used as blocks within which 20 × 20 m plots were nested. Removing singleton species did not alter the analyses output in any meaningful way, so we opted to show the results with all species. We illustrated compositional differences between years and management history categories through nonmetric multidimensional scaling (NMDS). We set the number of dimensions to 4 to keep the stress below 0.20 (McCune, Grace, & Urban, 2002). The NMDS was run with the “metaMDS” function of the “vegan” package (center = T, trymax = 100). Dimensionality was assessed by examining the change in stress (the rank correlation between the calculated similarity distances and the plotted distances) as a function of dimension while stepping down from a six‐ to one‐dimensional solution.

We tested the independence of the number of trees from functional group, year, and management history through a generalized linear model (GLM) with Poisson error. A full model was fitted first, followed by manual variable removal based on Akaike's information criterion and test of significance of the interaction term with ANOVA (Zuur et al., 2009). Species that were not ascribed to any of the previously mentioned ecological groups were attributed to one of them using the wood density and total height distributions percentiles provided by Souza et al. (2014). Species with maximum height ≤15 m were assigned to the Small Shade Tolerant group. Species with maximum height between 15 and 19 m and wood density <0.65 g/cm^3^ were assigned to the Pioneer group. Species with maximum height ≥19 m and wood density <0.65 g/cm^3^ were assigned to the Large Seeded Pioneer group. None of the previously unclassified species were assigned to the other groups. To assess plot‐level net changes over time in forest biomass, forest architecture, and trait values, we used ANOVA and bootstrapping methods (as in Feeley et al., 2011; Fauset et al., 2012).

#### 2.2.3 Forest structure and traits

Aboveground biomass was calculated separately for angiosperms, *Araucaria angustifolia*, for the palm *Syagrus romanzoffiana*, and tree ferns. Wood density values were obtained from (Chave et al., 2006) for 101 species (61% of our species, and 85% of our stems). Aboveground biomass was calculated for angiosperms using the pantropical model from Chave et al. (2014): Aboveground biomass = 0.0673 × (*ρD* ^2^ *H*)^0.976^, where *ρ* is wood density (g/cm^3^), *D* is diameter (cm), and *H* is height (m). Dry biomass was calculated for *Araucaria angustifolia* using the model from Sanquetta, Watzlawick, Schumacher, and DeMello (2003) in which Aboveground biomass = Trunk biomass + Live branches biomass, with Trunk biomass = (0.4141 × [111.7988 ‒15.5317*D* + 0.8544*D* ^2^ + 0.0180*D* ^2^ *H*]), and Live branch biomass = (0.3687 × [94.4247 ‒12.5807*D* + 0.3381*D* ^2^ + 0.0091*D* ^2^ *H*]). Dry biomass of the palm *Syagrus romanzoffiana* and of the tree ferns *Dicksonia sellowiana* and *Alsophila* sp. were estimated using the equations given by Tiepolo, Calmon, and Rocha Feretti (2002), in which Palm aboveground biomass = 0.3999 + 7.907*H*, and Tree Fern aboveground biomass = −4,266,348/(1−(2,792,284 exp[0.313677*H*])). Wood density values were obtained from Chave et al. (2006) for 101 species (61% of our species, and 85% of our stems). For species with no wood density records, genus averages were used, as suggested by (Chave et al., 2005). These corresponded to 28% of species and 4.9% of sampled stems. *Araucaria angustifolia* wood density (0.4475 g/cm^3^) was calculated as the average between the values from Chave et al. (2006) and de Mattos, Bortoli, Marchesan, and Rosot (2006).

We calculated the community‐level weighted means (CWM, Lavorel et al. (2008) and the Functional Dispersion index (a measure of functional diversity) of trait values using the “FD” package (Laliberté & Legendre, 2010). The Functional Diversity index is the weighted mean distance in the multidimensional trait space of individual species to the weighted centroid of all species, where weights correspond to the relative abundances of the species. It has the desirable properties of not being sensitive to outliers, considering species relative abundances, and being only weakly influenced by species richness (Laliberté & Legendre, 2010).

The annual rates of net change in biomass, basal area, tree density, and CWM of traits were calculated as (*x*_2_ − *x*_1_)/*t*, where *x*_1_ is the initial plot‐level value, *x*_2_ is the final plot‐level value and *t* is the census interval (9 years). To test whether the CWM of traits were different between management history categories, we used PERMANOVA with Euclidean distances and 5,000 permutations, since the data did not meet the MANOVA multivariate assumptions. To test whether the mean rate of net change of a variable was significantly different from zero across the 250 sampled plots, we randomly selected the 250 plots, 5,000 times with replacement, and calculated the mean rate of net change of the variable for each bootstrap. If the 95% confidence intervals (CI) from the distribution derived from the bootstrapped data did not overlap zero, we considered that the net change in that variable was significant at the *p* < 0.05 level (Fauset et al., 2012; Feeley et al., 2011).

We used generalized linear models to evaluate how aboveground biomass was influenced by management history, functional diversity (FD index), and the CWM of traits as explanatory variables. We ran subsets of the full GLM model using different combinations of the explanatory variables and ranked them based on the AICc. In order to keep the number of models manageable and still include all types of biomass drivers, we grouped the explanatory variables into four predictor sets: forest structure (basal area and tree density), functional diversity (the functional dispersion index), management history, and the trait CWMs. We then built models using single predictor sets, all additive combinations of predictors, and models in which management history interacted with all possible combinations of the other predictor sets. The full model including interactions of management history with other sets of predictors was:

Biomass = Management History × (Functional Diversity + Forest Structure + Trait CWMs).

The set of best models (models equally supported) were considered as those with ΔAICc ≤ 2 (Burnham & Anderson, 2002). The residuals of the set of best models were evaluated for spatial autocorrelation using spatial correlogram of Moran's *I* coefficients using 10 distance classes. No spatial structure was detected in the residuals (*I* = 0.00093, *p* = 0.372). Before running the GLM models, multicollinearity between explanatory variables was evaluated through variance inflation factor before analysis (seven variables retained, VIF < 3.0, Zuur, Ieno, & Elphick, 2010). In order to decide whether a random term was necessary, we compared the second‐order Akaike information criterion (AICc, used for small sample sizes) between a full model using generalized least squares without a random term (i.e., containing fixed terms only) and a mixed model using plot identity (10 different 1‐ha plots) as a random intercept term (Zuur et al., 2009). Both models were fit by maximizing the restricted log‐likelihood. The random effect in the mixed model accounted for the possible lack of independence of the biomass values estimated for the 20 × 20 m of plots within the 1‐ha plots. The AICc values were very close (1,285.684 and 1,285.774 for the fixed and the mixed models, respectively), and we thus discarded the mixed model since its increased complexity did not represent an improvement relative to the fixed model.

For the GLMs used to evaluate the biomass drivers, variables selected using the VIF were management history (categorical), functional dispersion index, subplot basal area, subplot tree density, and the CWMs for tree height, leaf size, SLA, and wood density. The excluded variables were the CWMs for crown depth and seed size. The second‐order Akaike information criterion (AICc) was calculated using the package MuMIn (Barton, 2018). The full model using generalized least squares without a random term (i.e., containing fixed terms only) was fit using the gls function of the nlme R package (Pinheiro, Bates, DebRoy, & Sarkar, 2017) and the mixed model was fit using the lme function of the same package. Both models were fit with option method = “REML.” The spatial correlogram of Moran's *I* coefficients was run using 10 distance classes and the correlog function of the pgirmess package (Giraudoux, 2018).

## 3 RESULTS

### 3.1 Climate trends

**Figure 2:**
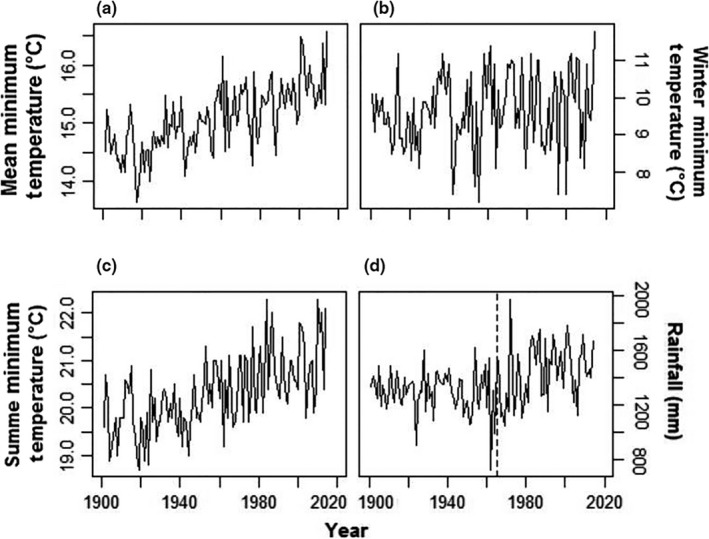
Annual mean minimum temperature (°C) (a) winter minimum temperature (°C) (b) annual mean summer temperature (°C) (c) and annual rainfall (mm) (d) from 1901 to 2014 at the 0.5 × 0.5° grid cell containing the eastern part of the *sul‐riograndense* highlands, where São Francisco de Paula and the studied forests are located in subtropical South America. The dashed line in the rainfall plot highlights 1965, the year from which a growing trend was detected in the data. Data from CRU (Harris et al., 2014)

**Figure 3:**
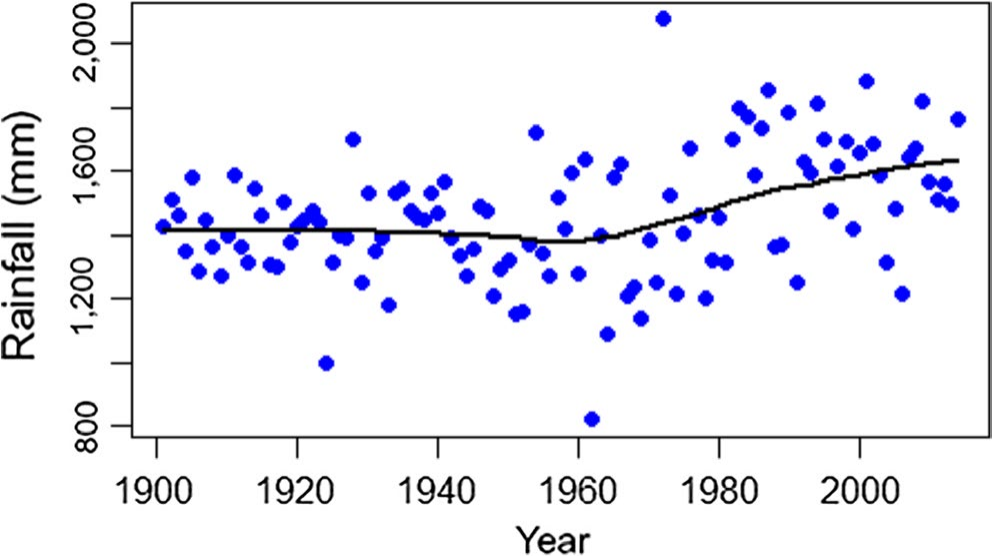
Loess curve fitted to annual rainfall data for the 0.5 × 0.5° grid cell containing the eastern part of the *sul‐riograndense* highlands, where São Francisco de Paula and the studied forests are located. The loess curve was fitted with *α* = 0.50, locally linear functional form (*λ* = 1), and robustness iterations

**Figure 4:**
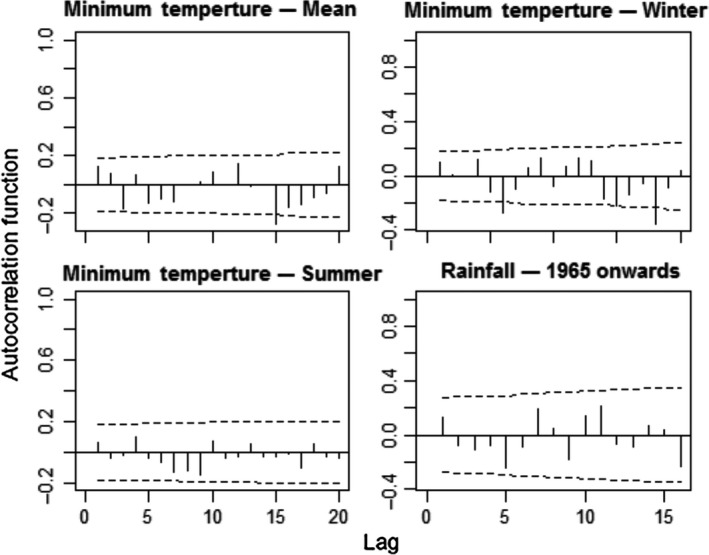
Autocorrelation functions of the residuals of linear regressions of annual mean, winter, and summer minimum temperatures and rainfall from 1965 onwards on sample year for the 0.5 × 0.5° grid cell containing the eastern part of the *sul‐riograndense* highlands, where São Francisco de Paula and the studied forests are located

Between 1901 and 2014, mean annual minimum temperatures increased significantly (Linear Regression, *F* = 100.8, *df* = 112, *p* < 0.0001, slope = 0.0118 ± 0.0018°C/year, Figure 2). This trend did not involve winter minimum temperatures, which did not show any tendencies through the same period (*F* = 2.9, *p* = 0.092). It was produced by summer minimum temperatures, which increased significantly (*F* = 64.3, *p* < 0.0001) by a mean of 1.49°C from the first (19.63 ± 0.54°C) to the last (21.12 ± 0.83°C) decade of the series. The annual rainfall series had two statistically significant extreme years (Grubbs test, *G* = 6.36, *p* = 0.017), which corresponded to a very dry (1962 with 821.3 mm) and a very rainy year (1972 with 2073.8 mm; Figure 2). A loess regression revealed an upward trend from 1965 onwards (Figure 3). Linear regressions confirmed this pattern, showing no trend from 1901 to 1964 (*F* = 1.84, *p* = 0.181) but an upward trend from 1965 to 2014 (*F* = 7.58, *p* = 0.008). On average, annual rainfall after 1965 (1,541 ± 214.14 mm) was 10.71% (149 mm) larger than in the years before 1965 (1,392 ± 154.78 mm; ANOVA, *F* = 18.47, *p* < 0.0001). None of the regressions were affected by temporal autocorrelation, as shown by residuals analyses (Durbin–Watson tests, *p* > 0.05, Figure 4).

### 3.2 Species composition and ecological groups

**Figure 5:**
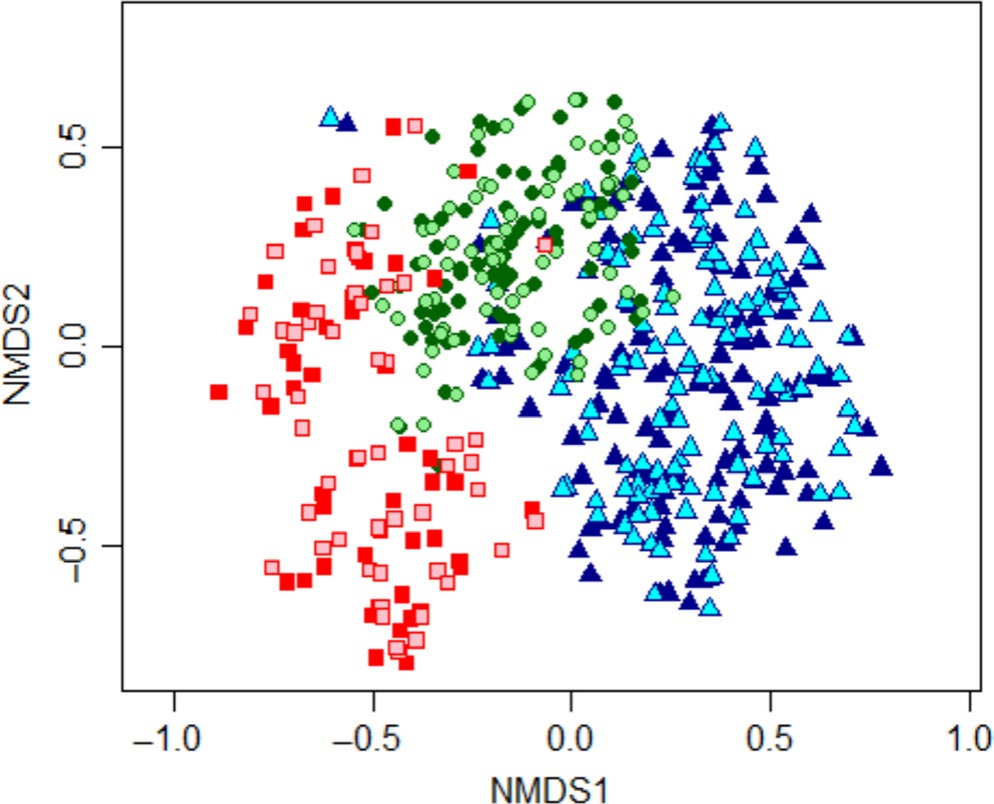
Nonmetric multidimensional scaling (NMDS) ordination of subtropical forest tree communities with distinct management histories in 2000 and 2009 in subtropical South America. Light symbols = 2000, Dark symbols = 2009, Δ = Unlogged plots, ᴏ = Early logged plots, □ = Recently logged plots

Tree species composition changed significantly from 2000 to 2009 (nested PERMANOVA, *F* = 0.847, *p* = 0.0002) although this change was a small one (*R* ^2^ = 0.0014) and was slightly different depending on management history (interaction term, *F* = 0.195, *p* = 0.0002, *R* ^2^ = 0.0007). The strongest compositional differences occurred between management histories (*F* = 50.42, *p* = 0.0002, *R* ^2^ = 0.169). Forests in each logging history category were floristically different from all others (pairwise PERMANOVA, *p* < 0.001 in all cases). The NMDS (stress = 0.177) clearly depicted the compositional difference between forests with distinct management histories (Figure 5). Unlogged plots were concentrated on the right side of the ordination plot, while recently logged forests were concentrated on the left side, and early‐logged forest plots lied in between. The clouds of points relative to 2000 and 2009 overlapped extensively.

**Figure 6:**
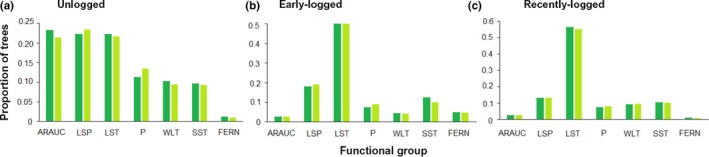
Proportions of trees belonging to different ecological groups in unlogged (a), early logged (b), and recently logged (c) forest stands in 2000 (dark bars) and 2009 (light bars). ARAUC, Araucaria; LSP, Large‐Seeded Pioneer; LST, Large Shade Tolerant; P, Pioneer; SST, Small Shade Tolerant; WLT, Wind‐Dispersed Large Tree

The compositional changes detected by the PERMANOVA were reflected in functional group abundances changes, which were also significant between years and between distinct management histories, with a significant interaction between these two explanatory factors. The final GLM model was the full model with the year:functional group:management history interaction (AIC = 389.00). The removal of the hither‐term interaction was significant (GLM Analysis of Deviance, Dev = −13,267, *p* < 2.2 × 10^−16^) and produced a much worse model (AIC = 13,574.16). Most of the abundance differences occurred between management history categories (Figure 6). Unlogged stands were dominated by Araucaria, Large Seeded Pioneers, and Large Shade Tolerants, with nearly equal abundances. Pioneers, Wind‐Dispersed Large Trees, and Small Shade Tolerants each corresponded to ca. 10% of the stems, while arborescent ferns were quite rare. However, in early‐logged and recently logged stands, functional structure was markedly diverse from the one found in unlogged areas, being dominated by Large Shade Tolerants. Arborescent ferns were more abundant in early‐logged stands than in either unlogged or recently logged ones. Temporal changes were minor and occurred mostly in unlogged stands, where the abundance of Araucaria suffered a small decline, while Pioneers and Large Seeded Pioneers increased.

### 3.3 Forest structure and traits

**Figure 7:**
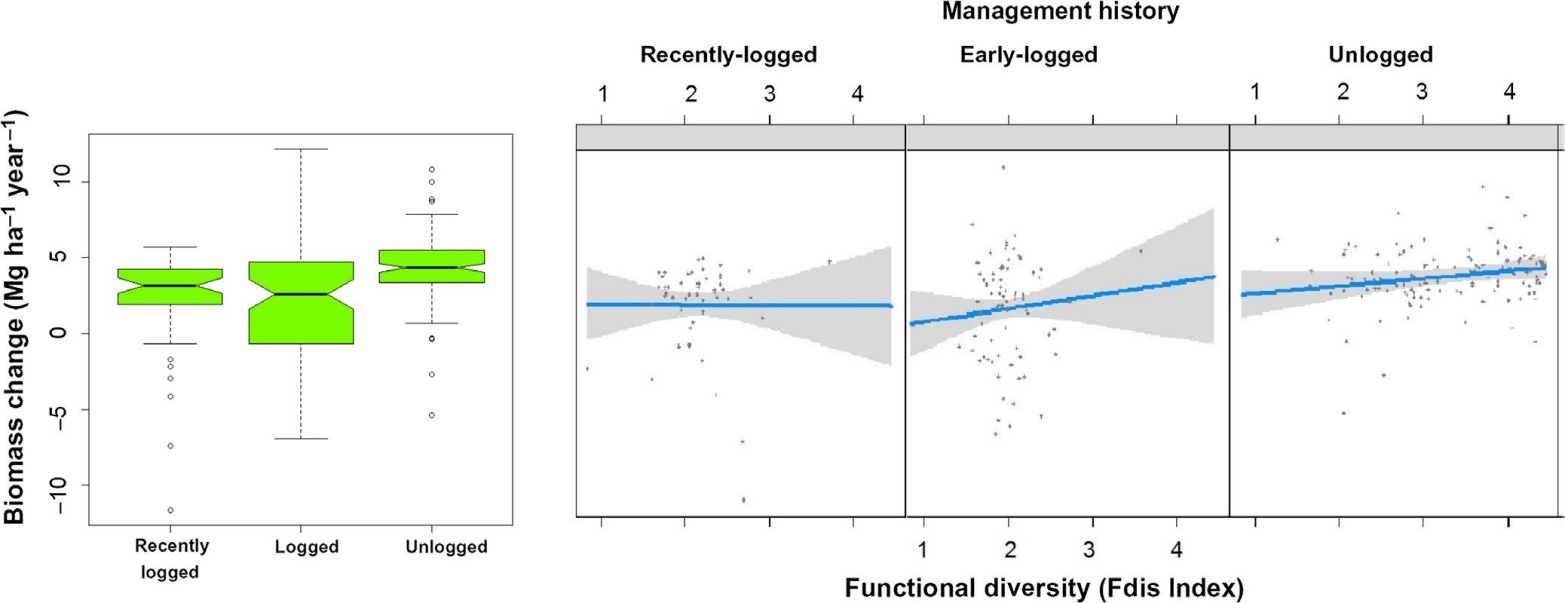
Biomass rate of net change in mixed subtropical forest stands subjected to different logging histories in subtropical South America. Biomass net change is shown both in forest patches with different management histories (left) and as a function of functional diversity (right). In the box plot, the central notches represent median values; upper and lower vertical bars (whiskers) are the fourth and first quartiles, respectively; notches surrounding the median represent median 95% confidence intervals; vertical bars connect the minimum and maximum values measured; o, extreme value; *, outlier. Nonoverlapping of notches indicates significant difference at 95% confidence level

Total aboveground biomass increased significantly during the 9‐year interval, growing from 337.21 ± 49.82 Mg/ha in 2000 to 366.66 ± 49.98 Mg/ha in 2009. Plot‐size mean increase was 3.27 ± 3.09 Mg/ha per year (bootstrapped 95% CI = 2.88–3.67 Mg/ha per year). Our model selection procedure yielded two GLMs relating biomass change with sets of explanatory variables whose ΔAICc ≤ 2 relative to the model with lowest AICc (Table 1). The three models contained management history alone, or management history in addition to or interacting with functional diversity (Figure 7). Unlogged stands showed significantly greater biomass increases (bootstrapped 95% CI = 4.01–4.74 Mg/ha per year) than early‐logged and recently logged stands (bootstrapped 95% CI = 1.21–2.91 Mg/ha per year, pooled data). Functional diversity was positively related to biomass increase in unlogged plots only. Basal area also increased significantly during the census interval with a plot‐size mean increase of 0.69 ± 0.47 m^2^/ha per year (bootstrapped 95% CI = 0.63–0.75 m^2^/ha per year), but was not different between logging histories (ANOVA, *F* = 1.68, *df* = 2, *p* = 0.19).

**Table 1:** Generalized linear models used to investigate the influence of management history (Man.H), functional diversity (Fdis), forest structure (subplot basal area and tree density), and the community‐weighted means of tree height, SLA, leaf size, wood density (Trait CWM). The three top models (those with the smallest AICc and whose ΔAICc ≤ 2) are in italics, while the best model is in bold letters. The best model was the simples of the top models

Model	AICc
Man. H*Fdis	1,246.802
*Man. H + Fdis*	1,246.874
***Man. H***	1,247.182
Man.H*Trait CWM	1,250.901
Fdis	1,253.141
Man. H + Trait CWM	1,257.624
Man. H + Forest Structure	1,260.634
Fdis + Trait CWM	1,262.183
CWMheigth	1,262.55
Trait CWM	1,267.595
Fdis + Forest Structure	1,271.168
CWMstem	1,277.698
CWMsla	1,278.484
CWMleaf	1,281.367
Man. H* (Trait CWM + Forest Structure + Fdis)	1,281.485
Forest Structure	1,282.358
Man. H* (Forest Structure)	1,287.407
Forest Structure	1,293.391

**Figure 8:**
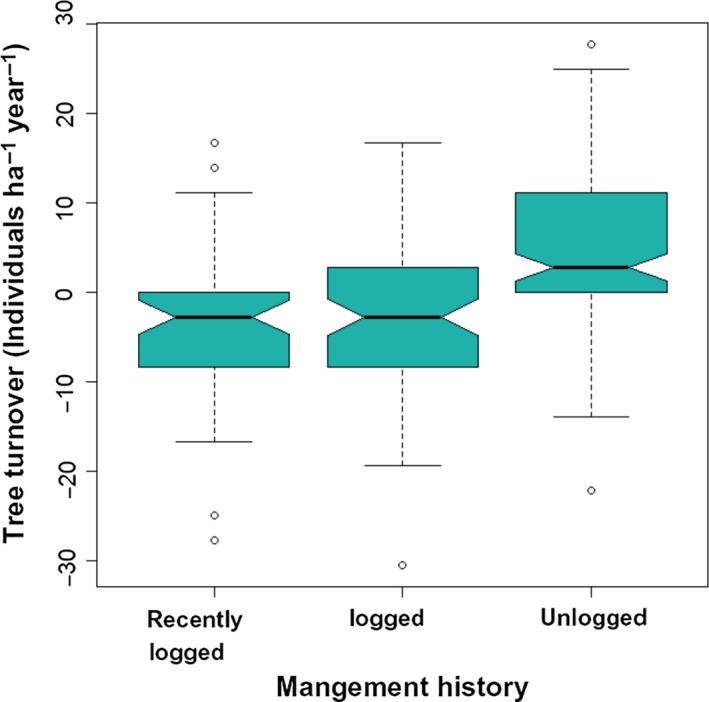
Tree turnover in mixed subtropical forest stands subjected to different logging histories in subtropical South America. Graphical elements are explained in Figure 7. Nonoverlapping of notches indicates significant difference at 95% confidence level

During the 9‐year census interval, overall stem density did not change significantly (initial value = 1,342.4 ± 446.27/stems ha in 2000; bootstrapped 95% CI change = −0.06–2.30 stems ha^−1^ year^−1^). However, this resulted from significantly different tendencies between forest stands with different logging histories (Figure 8, ANOVA, *F* = 21.48, *df* = 2, *p* < 2.5 × 10^−9^). Unlogged stands showed a significant mean increment of 4.76 stems ha^−1^ year^−1 ^(bootstrapped 95% CI = 3.23–6.29 stems ha^−1^ year^−1^), increasing from 1,160.80 ± 127.37 stems/ha in 2000 to 1,203.6 ± 97.21 stems/ha in 2009. Stem dynamics in early‐logged stands did not differ from zero (bootstrapped 95% CI = −3.88–0.17 stems ha^−1^ year^−1^). Recently logged stands, however, showed negative stem density net change (bootstrapped 95% CI = −6.17 to −0.83 stems ha^−1^ year^−1^), decreasing from a density of 1,750.00 ± 120.21 stems/ha in 2000 to 1718.50 ± 116.67 stems/ha in 2009.

**Figure 9:**
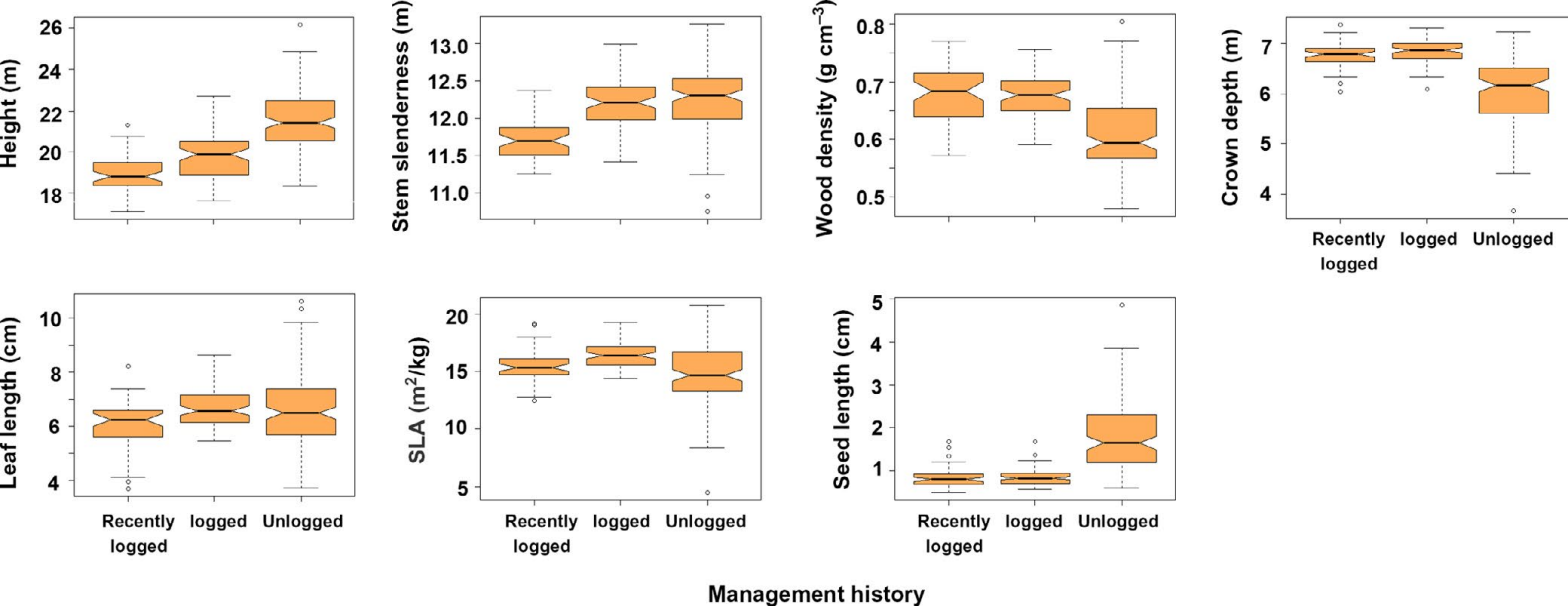
Community‐level weighted means of six functional traits in mixed subtropical forest stands subjected to different logging histories in subtropical South America in 2000. Graphical elements are explained in Figure 7. Nonoverlapping of notches indicates significant difference at 95% confidence level

**Figure 10:**
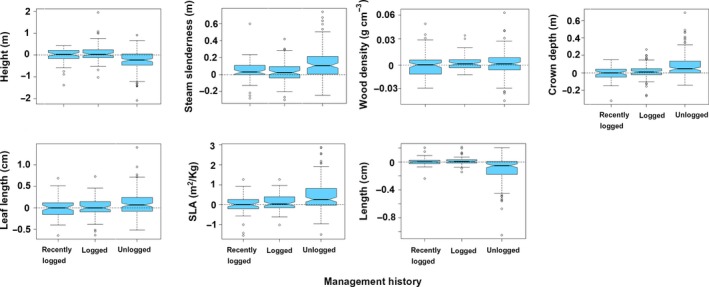
Changes in community‐level weighted means of six functional traits in mixed subtropical forest stands subjected to different logging histories in subtropical South America between 2000 and 2009. Graphical elements are explained in Figure 7. Nonoverlapping of notches indicates significant difference at 95% confidence level

**Figure 11:**
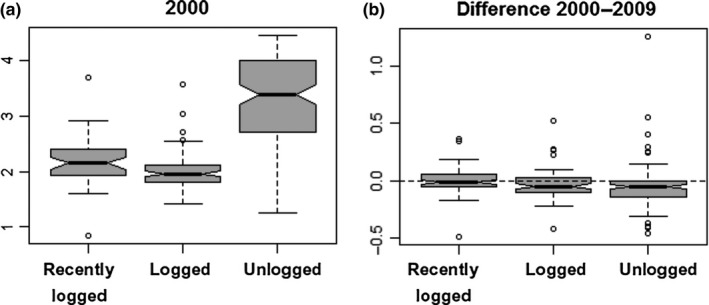
(a) The functional dispersion index in mixed subtropical forest stands subjected to different logging histories in subtropical South America in 2000, and (b) its change from 2000 to 2009. Graphical elements are explained in Figure 7. Nonoverlapping of notches indicates significant difference at 95% confidence level

The community‐weighted means of all traits were significantly different between management history categories (PERMANOVA, *F* = 30.13, *df* = 2, *p* = 2.0 × 10^−4^). In 2000, unlogged forest plots had taller trees, which had softer wood, more slender trunks and shallower crowns, and produced larger seeds (Figure 9). Leaves were smaller in recently logged plots, while SLA was larger in early‐logged plots. From 2000 to 2009, net changes in the CWM of these traits also differed significantly between management history categories (Figure 10, PERMANOVA, *F* = 11.25, *df* = 2, *p* = 2.0 × 10^−4^). The dynamics of trait CWM remained stationary in both logging management histories for all traits except for stem slenderness. This trait increased in modest amounts in early‐logged and recently logged plots but suffered significantly higher increases in unlogged plots. In contrast to logged plots, unlogged trait dynamics differed from zero in all studied traits. Besides increases in stem slenderness, unlogged forests presented significant increases in CWM crown depth, leaf length, and SLA, and significant decreases in height and seed size. In 2000, the functional dispersion index of early‐logged and recently logged plots was statistically similar (pooled CI: 2.00–2.14) and lower than the functional dispersion of unlogged plots (CI: 3.17–3.45; Figure 11a). Functional dispersion changes from 2000 to 2009 (Figure 11b) did not differ between stands with different logging histories (ANOVA, *F* = 3.07, *df* = 2, *p* = 0.081) and were weakly, although significantly, negative (CI of pooled data: −0.02 to −0.06).

## 4 DISCUSSION

Our results showed that noticeable functional changes took place in the Atlantic forests of Southern Brazil, despite only minor temporal changes at the taxonomic level. Through the evaluation of structural and trait changes between management history categories, coupled with analysis of regional climate trends, we were able to identify the most likely mechanisms underlying the observed changes. Our hypothesis that climate change affected forest structure and dynamics differentially depending on forest succession following past disturbances was confirmed. Aboveground biomass increased in all plots, but increases were higher in mature unlogged forests, which showed signs of forest growth associated with increased CO_2_, temperature, and rainfall/treefall gap disturbance at the same time. Logged forests showed arrested succession as indicated by reduced abundances of Pioneers and biomass‐accumulators like Large Seeded Pioneers and Araucaria, and, as well as reduced functional diversity. Furthermore, our regional climatic analyses illustrate the importance of understanding the regional climatic background of community‐ and landscape‐scale studies, which are frequently carried out under the unchecked and possibly unrealistic premise of stationary climatic conditions. Regional climate change may impact ecosystem processes like primary productivity (Lewis et al., 2013), and present idiosyncratic patterns that need to be understood in a case by case mode (IPCC, 2013).

### 4.1 The functional dynamics of mature forests

Our results supported the expectations that mature forest dynamics are driven by the combined action of increased CO_2_/temperature and increased rainfall/treefall gap dynamics. The biomass and basal area increases we found (0.69 m^2^/ha per year) were higher than those found in subtropical deciduous forests in Argentina (0.13 m^2^/ha per year, Malizia et al., 2013) and in 50 plots distributed across tropical South America from a 30‐year period (0.10 m^2^/ha per year, 1971–2002; Lewis et al., 2004). The fact that basal area increase did not differ between forests with different logging histories is strong evidence of a pervasive resource level increase like rising CO_2_ atmospheric concentration, as has been inferred based on pantropical plot networks (Holtum & Winter, 2010; Lewis et al., 2009, 2004; Phillips et al., 2016). A number of factors are likely contributing to the very high aboveground biomass and basal area accumulation we observed. One of these is the rising minimum temperatures we detected. Low air temperatures constitute a limiting factor for photosynthesis and biomass increase (Lewis et al., 2013) and, in the subtropics, they constitute a limiting factor for tree growth (Oliveira et al., 2010; Zanon & Finger, 2010).

A second factor that helps explain the increased biomass accumulation in unlogged forests is the presence of very large trees. Density of large trees explains most of the variation in pan‐tropical aboveground biomass, and is positively associated with rainfall and temperature (Slik et al., 2013). Mature mixed Atlantic forests are dominated by large trees, mostly massive araucarias that accumulate high biomass (Chassot, Fleig, Finger, & Longhi, 2011; Rosenfield & Souza, 2014). The presence of this species, with its' large size, abundance, but light wood (Sanquetta et al., 2003), explains the apparent paradox of unlogged forests, with all its araucarias left intact and higher biomass, nevertheless presented reduced CWM wood density compared to logged stands. Araucaria's growth is limited by atmospheric CO_2_ levels and low temperatures (Cattaneo, Pahr, Fassola, Leporati, & Bogino, 2013; Oliveira et al., 2010; Silva, Anand, Oliveira, & Pillar, 2009). Therefore, climate change in subtropical wet South America has been highly favorable for biomass accumulation by large trees, and to araucarias in particular.

There was also evidence that unlogged forests suffered increased treefall gap disturbance rates. The increased rainfall we detected and a tornado that passed through the study region in 2003 (Candido, 2012) agree with the general climatic trend of increasing rainfall in subtropical South America since the mid‐20th century (IPCC, 2013) and peaking tornados and rainstorms in southern Brazil (Candido, 2012). This scenario supports our Increased Rainfall/Treefall Gap hypothesis in that increased rainfall would result in increased small scale disturbance events in the form of treefall gaps (Ge et al., 2013). As expected, windthrow mortality was highest among the tallest trees, concentrated in the Wind Dispersed Large Trees and Araucaria ecological groups (Beckert et al., 2014; Ebling et al., 2014), which declined in abundance. The increase of Pioneers and Large‐Seeded Pioneers we observed corresponds to increased regeneration opportunities in treefall gaps (Chami et al., 2011). It agrees with the well‐documented replacement of species groups during gap‐phase dynamics from stress‐tolerant species adapted to shade (high wood density, slow growth, large seeds, shallow crown) to competitive species adapted to resource‐rich environments (lightwood, fast‐growing, large leaves with high SLA, deep crowns, small seeds)(Wright et al., 2010; Grime & Pierce, ; Muscolo et al., 2014; Forgiarini, Souza, Longhi, & Oliveira, 2015, but see Falster et al., 2018; Wills et al., 2018).

The decline in the relative abundance of araucarias also agrees with their long‐lived pioneer ecological strategy and dependence on large‐scale disturbances for successful regeneration (Paludo, Lauterjung, dos Reis, & Mantovani, 2016; Souza, 2007; Souza et al., 2008). This regeneration niche was not met by the apparently small scale of treefall gap dynamics. The effects of the predicted increase in precipitation extremes in subtropical regions during the 21st century (IPCC, 2013) are uncertain. On the one hand, they may reduce some of the biomass accumulation potential of these forests through increased mortality of the largest trees and recruitment of younger and fast‐growing species in treefall gaps, as we and others have observed (Ge et al., 2013; Hogan et al., 2018). On the other hand, the main biomass builder of this system, which is *Araucaria angustifolia*, showed historical increase in response to increased wetness in the last 1,100 years (Behling, Pillar, Orlóci, & Bauermann, 2004).

Contrary to our prediction, the abundance of the light‐limited Small Shade Tolerant trees did not change in unlogged stands during the study period. This probably resulted from the cancelation of the positive effects of rising CO_2_, temperature, and light levels near treefall gaps (Muscolo et al., 2014) by light reductions due to increased cloud cover and higher mortality rates due to falling trees and debris during rainstorms (Forgiarini et al., 2015; Lewis et al., 2013; Uriarte et al., 2016). We attribute the restriction of these patterns to unlogged forest stands to the lack very large trees prone to windthrow in logged stands (Chazdon, 2003; Souza et al., 2012) also reported that a cyclone in Australia impacted an old‐growth forest but not a logged forest.

### 4.2 The functional dynamics of logged forests

The ecological groups structure and the functional dynamics in logged forests were markedly different from unlogged ones. On the one hand, logged forests showed clear signs of recovery from past disturbances. In recently logged forests, where selective timber extraction occurred until the 1980s, aboveground biomass and basal area increased, while tree density decreased. These structural changes fit the well‐known secondary succession trajectory from low biomass and dominance by young trees, through increased mortality produced by self‐thinning, to late successional forests with lower density and larger trees (Lewis et al., 2013; Muscolo et al., 2014; Stephenson & Mantgem, 2011). Furthermore, Pioneers declined in abundance while the CWM of stem slenderness increased, as typically happens during successional changes where fast‐growth, resource acquiring species are replaced by slow growth, shade‐tolerant species (Edwards et al., 2014; Grime & Pierce, ; Poorter et al., 2003). The fact that management history was the most constant predictor explaining biomass change in our GLMs agrees with Pyles et al. (2018), who found that disturbance history had an overwhelming influence on Atlantic Forest tree biomass irrespective of functional diversity and trait CWM at regional scales in eastern Brazil. Importantly, the restriction of positive relationship between biomass change and functional diversity to unlogged plots likely results from complementary effects between angiosperms and araucaria. Indeed, araucaria has been shown to present a distinct niche (Souza, Bezerra, & Longhi, 2016), set of trait values (Souza et al., 2014), and regeneration strategy (Souza, 2007; Souza et al., 2008) compared to angiosperm species, and accumulate much of the aerial biomass of conserved mixed forests (Rosenfield & Souza, 2014). This points to niche complementarity in the exploration of available resources between these two functional groups, what has been observed to lead to increased ecosystem performance like biomass accumulation (Loreau & Hector, 2001).

Despite signs of structural maturation, floristically and functionally logged forests showed signs of arrested succession. Souza et al. (2012) had already detected that species composition of logged forests differed from unlogged ones, and Souza et al. (2016) found that the intensity of past logging events was a stronger structuring factor for species distributions in the region than abiotic factors like rock cover, elevation, and soil drainage, as has been found elsewhere (Both et al., 2019). The compositional differences between stands with different logging histories corresponded to a marked reduction in the densities of araucarias, Large Seeded Pioneers, Wind Dispersed Large Trees, and Pioneers in logged forests in relation to unlogged stands, even after more than 60 years of cessation of logging operations. These groups included long‐lived pioneers that need large‐scale disturbances to recruit to adult sizes (Enright, Ogden, & Rigg, 1999; Souza et al., 2014). Together with the interruption of araucaria's regeneration pulse in early‐logged stands (Souza et al., 2008), this indicates that selective logging was not disruptive enough to allow adult tree replacement. The lack of long‐lived pioneers and Wind Dispersed Large Trees in logged forests explains their higher community‐weighted wood density (araucaria is a light‐wooded species) and the reduced community‐weighted height and seed size (Forgiarini et al., 2015; Grime & Pierce, ; Muscolo et al., 2014; Wright et al., 2010). Uncontrolled logging can cause significant forest degradation (Chazdon, 2003; Edwards et al., 2014; Longo & Keller, 2019), recruitment shifts between ecological groups (Hogan et al., 2018), and chronic regeneration failure of exploited long‐lived pioneers (Enright et al., 1999; Peña‐Claros et al., 2008; Souza, 2007; Souza et al., 2008; Ter Steege, Welch, & Zagt, 2002).

In an intermediate position between unlogged and recently logged stands, the dynamics of early‐logged forests seemed to be dominated by a combination of both recovery from past disturbances and increased rainfall/treefall gap dynamics. As in unlogged forests, the increased basal area and aboveground biomass of early logged forests could be partly caused by increasing CO_2_ and temperature, global climatic processes that have been shown to affect forests worldwide (Lewis et al., 2009; Phillips et al., 2016; Poorter et al., 2017). However, these forests did not show any changes in tree density, and their overall structure, including sapling and pole densities, approached that of unlogged stands (Souza et al., 2012). Their community‐weighted leaf length and crown depths approached the levels found in unlogged forests, while their reduced CWM seed size and height, and increased crown depth and SLA indicate functional relevance of fast‐growth resource‐acquiring strategies (Forgiarini et al., 2015; Grime & Pierce, ; Wright et al., 2010), although SLA may not be much influential for canopy trees (Falster et al., 2018; Wills et al., 2018). The logging of Wind Dispersed Large Trees and the long‐lived pioneers probably set subtropical Atlantic forests on a successional trajectory that proceeds without these functional groups (Edwards et al., 2014). Logged forests are growing with angiosperm dominance instead of the typical araucaria dominance, and lack other common taxa like *Ilex paraguariensis* and *Maytenus aquifolia* (Gonçalves & Souza, 2014; Souza et al., 2012).

### 4.3 An overall picture and global implications

Our results represent one of the few detailed accounts of the dynamics of well‐conserved stands of South American subtropical rainforests (see also Pizatto 1999, Castanho et al., 2015). Authors working in subtropical Brazil (Ebling et al., 2014; Formento, Schorn, & Ramos, 2004; Castanho et al., 2015; Pizatto, 1999) also did not find major compositional changes in the mature forests they studied. These findings agree with Souza et al. (2016), who found that the species composition of the same forests we studied matches patterns generated by quasi‐neutral dynamics, an assembly mechanism intermediate between purely neutral and niche, with a high degree of stochasticity and drift overlaid on environmental affinities. Our study also highlighted that the effects of human impacts on forest changes can vary substantially between regions. Climate change may reduce forest biomass stocks in many tropical regions due to reduced rainfall and increased prevalence of fast‐growing drought‐adapted generalist species with lighter wood, smaller seeds, and smaller adult sizes (Enquist & Enquist, 2011; Lewis et al., 2009; Poorter et al., 2017; Toledo et al., 2011). Contrary to this trend, our data suggest that subtropical mixed rainforests could represent an exceptional global carbon sink, with very high biomass accumulation rates due to the combination of global fertilization effects of rising CO_2_ concentrations with regionally rising but not damaging temperatures (Lewis et al., 2009), lack of water shortage (IPCC, 2013), and presence of massive conifers. The aboveground carbon stock of such forests (125.1 Mg/ha) has been estimated to be twice as large as that of semideciduous forests in the same region (Rosenfield & Souza, 2014). The reduction of these forests to ca. 13% of their original 3,202,134 ha extension (Ribeiro, Metzger, Martensen, Ponzoni, & Hirota, 2009), coupled with pervasive selective logging of abundant and large‐sized araucarias, represents a significant source of CO_2_ release into the atmosphere.

Treefall gap disturbances and trait‐mediated secondary succession in forest ecosystems are ubiquitous (Grime & Pierce, ; Lohbeck et al., 2014; Muscolo et al., 2014; Pickett et al., 1989) and important for the maintenance of native plant communities (Davies et al., 2009). Yet, it is likely that the response of plant communities to them depend on other disturbances like climatic change, as argued by Davies et al. (2009). Although unsustainable selective logging represents a milder disturbance type than other forms of human activity (i.e., agriculture), it can lead to long‐lasting functional (Both et al., 2019) and biodiversity degradation of logged populations (Chazdon, 2003; Edwards et al., 2014; Souza et al., 2012; Ter Steege, Welch, & Zagt, 2002). In wet subtropical South America, logging altered CWMs of several traits that are important for forest productivity and functioning like SLA, wood density, and seed size (Díaz & Cabido, 2001; Díaz et al., 2016; Forgiarini et al., 2015). Logging reduced the overall functional diversity relative to unlogged forests, and this reduction did not show signs of recovery after more than 60 years logging operations stopped. This functional impoverishment was due to the lack of natural regeneration of ecological groups targeted by logging, mainly long‐lived pioneers that depend on large‐scale disturbance and slow growing and large‐sized Wind Dispersed Large Trees. Our results suggest that active management is therefore needed in order to restore the full functionality and ecosystem services like increased aboveground biomass accumulation in large tracts of logged subtropical mixed Atlantic forests in Southern Brazil and Argentina, since long‐lived pioneers like Araucaria and Large‐Seeded Pioneers will probably not recover their typical dominance in these forests by natural succession. We suggest the creation of planned canopy openings in order to create regeneration opportunities for the long‐lived pioneers (Peña‐Claros et al., 2008), accompanied by the direct plantation of species of this group in logged forests when surrounding seed sources are not available.

## CONFLICT OF INTEREST

The authors have no conflict of interest to declare.

## AUTHOR CONTRIBUTION

Solong J. Longhi planned the sampling design and collected the data. Alexandre F. Souza conceived the study, analyzed the data, and wrote the paper.

## Data Availability

Climate, biomass, and forest structure data are available in Dryad: https://doi.org/10.5061/dryad.438t5kg.
